# First identification of an IMI-1 carbapenemase-producing colistin-resistant *Enterobactercloacae* in China

**DOI:** 10.1186/s12941-015-0112-2

**Published:** 2015-11-25

**Authors:** Liang Huang, Xiaohui Wang, Yu Feng, Yi Xie, Liping Xie, Zhiyong Zong

**Affiliations:** Center of Infectious Diseases, West China Hospital (Huaxi), Sichuan University, Guoxuexiang 37, 610041 Chengdu, China; Division of Infectious Diseases, State Key Laboratory of Biotherapy, Chengdu, China; Laboratory of Clinical Microbiology, Department of Laboratory Medicine, West China Hospital, Sichuan University, Chengdu, China; Department of Haematology, West China Hospital, Sichuan University, Chengdu, China

**Keywords:** β-Lactamases, Carbapenemases, Resistance plasmids, *bla*_IMI_, *Enterobacter cloacae*

## Abstract

**Background:**

Carbapenem resistance among the Enterobacteriaceae is a serious healthcare challenge. *bla*_IMI_ is a carbapenemase gene mediating resistance to carbapenems but has not been commonly found. A *bla*_IMI_-carrying *Enterobactercloacae*, which was also resistant to colistin, is reported here.

**Findings:**

*E. cloacae* strain WCHECl-1060 was recovered from a blood sample of a leukemia patient, who was not previously exposed to colistin. Strain WCHECl-1060 belongs to a new sequence type, ST410, and was resistant to carbapenems and colistin but was susceptible to third-generation cephalosporins. A new allelic variant of *bla*_IMI-1_, which has two silent mutations compared to the original *bla*_IMI-1_ variant, was found in strain WCHECl-1060. Conjugation and transformation experiments failed to transfer *bla*_IMI-1_, suggesting a likely chromosome origin.

**Conclusions:**

To our knowledge, this is the first report of an IMI-1 carbapenemase-producing colistin-resistant *E.cloacae* in China. Microbiological laboratories should be aware of the unusual carbapenem-resistant but third-generation cephalosporin-susceptible profiles of these IMI-producing isolates. The trend of colistin resistance among the Enterobacteriaceae should be also monitored.

## Findings

*Enterobacter cloacae* strain WCHECl-1060 was recovered from the blood of a 30-year-old male patient with acute lymphoblastic leukemia in our hospital on October 2014. Species identification was performed using the Vitek II automated system (bioMerieux, Lyon, France) and was also confirmed by partially sequencing the *gyrB* gene as described previously [1]. In vitro susceptibility tests were performed using the Vitek II system and minimum inhibitory concentrations (MICs) of amikacin, ceftazidime, ciprofloxacin, colistin, imipenem and tigecycline were also determined using the microdilution broth method followed recommendations of the Clinical Laboratory Standards Institute [2]. Strain WCHECl-1060 was susceptible to ceftazidime (MIC 1 μg/mL), amikacin (MIC 4 μg/mL), ciprofloxacin (MIC 8 μg/mL) and tigecycline (MIC 4 μg/mL) but resistant to imipenem (MIC 256 μg/mL) and colistin (MIC >256 μg/mL). Strain WCHECl-1060 was also susceptible to aztreonam, ceftriaxone, cefepime, piperacillin–tazobactam, gentamicin, tobramycin, levofloxacin, trimethoprim–sulphamethoxazole and minocycline, intermediate to nitrofurantoin and cefotetan, and resistant to meropenem and ertapenem as determined by Vitek II.

Strain WCHECl-1060 was screened using PCR for *bla*_IMI_ with in-house designed primers IMI-Fn (AGAGTTCYATTCACCCATCACA) and IMI-Rn (TCTCCAATCGACCGCATGAA) and for other acquired carbapenemase-encoding genes *bla*_GES_, *bla*_KPC_, *bla*_IMP_, *bla*_NDM_, *bla*_OXA-48_ and *bla*_VIM_ as described previously [3–6]. *bla*_IMI_ was the only carbapenemase gene detected in strain WCHECl-1060. The complete coding sequence of *bla*_IMI_ was further amplified with an additional pair of in-house designed primers, IMI-up (CTGGCACGCATAGTAACCCA) and IMI-dw (ATGCCGAAAGTGCAAGCCT). Sequencing of the amplicon revealed the presence of *bla*_IMI-1_. Of note, the *bla*_IMI-1_ gene identified here had two silent mutations (T201C and C459T, positions assigned with respect to the ATG start codon of the *bla*_IMI-1_ gene) compared to the original *bla*_IMI-1_ gene (GenBank accession number U50278) found in the USA and has one silent mutation (T201C) to another *bla*_IMI-1_ variant (GenBank accession JX090311) found in Taiwan. In total, three types of *bla*_IMI-1_ have been found so far and the *bla*_IMI-1_ gene identified here is a new variant whose sequence has been deposited into the GenBank under the accession number KP284436.

Strain typing was performed using the multi-locus sequence typing (MLST) scheme for *E. cloacae* (http://pubmlst.org/ecloacae/). Strain WCHECl-1060 was of a new sequence type, ST410, which was assigned by the curator of the MLST database.

Conjugation experiments were performed using the azide-resistant *Escherichia coli* strain J53 as the recipient with potential transconjugants being selected on media containing 2 μg/mL imipenem and 150 μg/mL sodium azide. Transformation experiments were carried out using plasmid DNA prepared from WCHECl-1060 by alkaline lysis, which was electroporated into *E. coli* strain DH5α with potential transformants being selected on media containing 2 μg/mL imipenem. Both conjugation and transformation experiments failed to transfer carbapenem resistance, suggesting that *bla*_IMI-1_ was likely located on the chromosome. This result was not surprising considering that most *bla*_IMI_ genes found so far are chromosome-borne [7–9].

Currently, there are 11 assigned variants of IMI enzymes (IMI-1 to -11, http://www.lahey.org/Studies/other.asp#table1). In addition, carbapenemase NCM-A shares 97 % amino acid identity with IMI-1 and is therefore also a type of IMI enzyme. The distribution of IMI-producing isolates is summarized in Table 1. Two features of the distribution of IMI-producing isolates could be noticed. First, although IMI/NCM-A type enzymes have been reported for 20 years, surprisingly, only a few IMI-producing isolates have been identified since then. In contrast, another Class A carbapenemase, KPC, has been commonly found in carbapenem-resistant Enterobacteriaceae worldwide. Second, although IMI-producing isolates are not common, they have a wide geographic distribution having been found in isolates from Asia, Europe, and North and South Americas (Table 1). Several reasons could be proposed for the scarce identification of IMI-producing isolates. IMI has only weak activity against third-generation cephalosporins [7] and, based on available data, IMI-producing *Enterobacter* spp. are also usually susceptible to ciprofloxacin [10, 11]. As third-generation cephalosporins and fluoroquinolones are the most common antimicrobial agents used for treating human infections, the susceptibility of IMI-producing isolates to these commonly-used agents may hinder their wide dissemination. In addition, genes encoding IMI-1 and NCM-A are largely found on the chromosome rather than carried by plasmids, which may serve as an obstacle for horizontal transfer of the genes between strains and between species. Nonetheless, the current epidemiological pattern of IMI-producing strains have not been fully understood and therefore more surveillances for IMI-producing strains is warranted. The exact origin of IMI enzymes and how it emerged in *Enterobacter* spp. also remain unknown.

**Table 1:** The distribution of IMI-producing isolates

IMI type^a^	Host species	Location	Accession number	References
1	*E. cloacae*	USA	U50278	[7]
	*E. cloacae*	Taiwan	JX090311	
	*E. cloacae*	France		[8]
	*E. asburiae*	Ireland		[10]
	*E. cloacae*	Canada	KR057494	
	*E. cloacae*	China		This study
2	*E. coli*	Spain	JN412066	[15]
	*E. asburiae*	USA	DQ173429	[16]
	*E. cloacae*	China	AY780889	[12]
3	*E. cloacae*	Hong Kong	GU015024	[11]
4	*E. cloacae*	Singapore	KF958750	
7	*E. cloacae*	Singapore	KM103296	
8	*E. cloacae*	UK	KP081315	
NCM-A	*E. cloacae*	Argentina	AJ536087	
	*E. cloacae*	Canada	KR057492	
	*E. cloacae*	Canada	KR057493	
	*E. cloacae*	Canada	KR057495	
	*E. cloacae*	Canada	KR057496	
	*E. cloacae*	France	Z21956	[9]

^a^ *bla*_IMI-5, -6, -9, -10 and -11_ have been assigned but no sequences nor publications are available

This is the first report in China of an *E. cloacae* clinical isolate that carries *bla*_IMI-1_ and is resistant to colistin. Nonetheless, three IMI-producing *E. cloacae* strains have previously been found in China. IMI-1 was found in Taiwan (GenBank accession number JX090311) but no details such as susceptibility are available for the host strain. IMI-2 was detected in a single strain in Zhejiang Province and IMI-3 was reported from Hong Kong but the susceptibility of both strains to colistin has not been tested [11, 12].

The resistance to colistin of strain WCHECl-1060 is unexpected and worrying as colistin is not available for clinical treatment in China, the patient had no exposure to this agent and *Enterobacter* spp. is not intrinsically resistant to colistin [13]. Unfortunately, there is no national data on the susceptibility of *Enterobacter* spp. to colistin in China. As colistin is expected to become available in China soon, monitoring resistance to colistin among the Enterobacteriaceae is required at both national and local levels. Resistance to colistin imposes a great challenge for treating carbapenem-resistant Enterobacteriaceae. However, the mechanism conferring resistance to colistin among *Enterobacter* spp. remains largely uninvestigated although a recent study revealed resistance to colistin in an *E. cloacae* strain recovered in the USA is associated with cross-resistance to the host antimicrobial lysozyme [14]. Further investigation for the resistance mechanism of strain WCHECl-1060 is warranted.

In conclusion, this is the first report in China of an IMI-producing colistin-resistant *E. cloacae* clinical isolate. The unusual resistance profile, i.e. being resistant to carbapenems while susceptible to third-generation cephalosporins, of the Enterobacteriaceae should prompt laboratories to be aware of the presence of IMI enzymes. As colistin is crucial last resort antimicrobial against infections caused by Gram-negative bacilli, monitoring resistance to colistin among *Enterobacter* spp. and studies on the colistin-resistance mechanism are much needed to combat antimicrobial resistance.

### Availability of supporting data

The sequence of the *bla*_IMI-1_ variant in this study has been deposited into the GenBank under the accession number KP284436. The MLST profile of strain WCHECl-1060 is available at the *E. cloacae* MLST database (http://pubmlst.org/ecloacae/).

## Abbreviations

MICs: minimum inhibitory concentrations
MLST: multi-locus sequence typing
